# SH3‐class Ras guanine nucleotide exchange factors are essential for *Aspergillus fumigatus* invasive growth

**DOI:** 10.1111/cmi.13013

**Published:** 2019-02-15

**Authors:** Adela Martin‐Vicente, Ana Camila Oliveira Souza, Qusai Al Abdallah, Wenbo Ge, Jarrod R. Fortwendel

**Affiliations:** ^1^ Department of Clinical Pharmacy and Translational Science University of Tennessee Health Science Center Memphis Tennessee USA

**Keywords:** aspergillosis, *Aspergillus fumigatus*, germination, guanine nucleotide exchange factor, polarity, Ras, virulence

## Abstract

Proper hyphal morphogenesis is essential for the establishment and progression of invasive disease caused by filamentous fungi. In the human pathogen *Aspergillus fumigatus*, signalling cascades driven by Ras and Ras‐like proteins orchestrate a wide variety of cellular processes required for hyphal growth. For activation, these proteins require interactions with Ras‐subfamily‐specific guanine nucleotide exchange factors (RasGEFs). Although Ras‐protein networks are essential for virulence in all pathogenic fungi, the importance of RasGEF proteins is largely unexplored. A. fumigatus encodes four putative RasGEFs that represent three separate classes of RasGEF proteins (SH3‐, Ras guanyl nucleotide‐releasing protein [RasGRP]–, and LTE‐class), each with fungus‐specific attributes. Here, we show that the SH3‐class and RasGRP‐class RasGEFs are required for properly timed polarity establishment during early growth and branch emergence as well as for cell wall stability. Further, we show that SH3‐class RasGEF activity is essential for polarity establishment and maintenance, a phenotype that is, at least, partially independent of the major A. fumigatus Ras proteins, RasA and RasB. Finally, loss of both SH3‐class RasGEFs resulted in avirulence in multiple models of invasive aspergillosis. Together, our findings suggest that RasGEF activity is essential for the integration of multiple signalling networks to drive invasive growth in A. fumigatus.

## 1 INTRODUCTION

Invasive aspergillosis (IA), mainly attributed to the species *Aspergillus fumigatus*, is frequently associated with high morbidity and mortality rates in immunocompromised patients, including those receiving bone marrow and solid organ transplants (Patterson et al., 2016). Once inhaled, the conidia of A. fumigatus must germinate and establish hyphae, highly polarised growth forms that invade and destroy tissues and organs. Filamentous fungal pathogens, like A. fumigatus, need to coordinate multiple cellular processes to affect hyphal morphogenetic processes and achieve tissue invasion. A wealth of published data has revealed that genetic mutations debilitating these processes leads to significant reductions in virulence (Abad et al., 2010). Therefore, a deeper understanding of how fungi regulate polarised growth, both during the establishment and the maintenance of polarisation, carries the promise of identifying novel antifungal therapeutics.

In all major human and plant pathogenic fungi, signalling networks controlled by small GTPase proteins are integrated and synchronised to orchestrate proper hyphal morphogenesis (Fortwendel, 2012). For example, in the human pathogenic basidiomycete yeast *Cryptococcus neoformans,* Ras and Ras‐like proteins are required for hyphal development, mating, actin polarisation to sites of bud formation, and growth at physiological temperatures (Alspaugh, Cavallo, Perfect, & Heitman, 2000; Ballou, Selvig, Narloch, Nichols, & Alspaugh, 2013; Vallim, Nichols, Fernandes, Cramer, & Alspaugh, 2005). As a consequence, deletion of the major Ras gene, *RAS1*, results in avirulence in animal models of cryptococcosis (Alspaugh et al., 2002). The most common fungal infection, invasive candidiasis, is also dependent upon active Ras proteins as the yeast pathogen Candida albicans requires regulatable Ras pathway activity to generate invasive hyphal forms (Leberer et al., 2001). Similar to C. neoformans, expression of an activated form of C. albicans Ras1p promotes the transition from yeast to hyphae (Feng, Summers, Guo, & Fink, 1999; Leberer et al., 2001). In the major human pathogenic mould, A. fumigatus, exact spatiotemporal control of Ras protein activity is essential to both the establishment and maintenance of hyphal growth and for virulence in animal models of IA (Fortwendel et al., 2008; Fortwendel et al., 2012). Together, these previous findings suggest that the regulation of Ras‐controlled networks may represent important processes that, if disrupted by novel therapeutics, could result in beneficial outcomes for the host.

In pathogenic fungi, Ras proteins are localised mainly to the plasma membrane after post‐translational modification of the carboxy‐terminal CAAX motif by farnesylation and subsequent palmitoylation. This localisation is critical for Ras contribution to virulence in C. albicans, C. neoformans, and A. fumigatus likely through placing Ras in context with its cognate GTPase‐activating proteins (GAPs) and guanine nucleotide exchange factors (GEFs) as well as proper downstream effectors (Fortwendel et al., 2012; Piispanen et al., 2011). Interactions with GAP and GEF proteins are essential to drive transitions between the active (GTP‐bound) and inactive (GDP‐bound) states of Ras and loss of these interactions can have detrimental effects to normal growth in the pathogenic fungi. For example, studies in C. albicans show that cells lacking the Ras1 GAP, IRA2, are hyper‐filamentous and more sensitive to heat stress, whereas knockouts in the main Ras‐subfamily‐specific guanine nucleotide exchange factor (RasGEF), CDC25, are hypo‐filamentous (Enloe, Diamond, & Mitchell, 2000; Roemer et al., 2003). Although little is known about how GAP and GEF proteins are activated and to which external and internal signals they respond in fungal pathogens, a recent in‐depth study revealed that Ras1 activation in C. albicans is modulated in response to changes in mitochondrial output. This was shown to be accomplished through adenylate cyclase‐mediated upregulation of the Ras1 GAP, Ira2p, in response to cellular ATP availability but was independent of the Ras GEF, Cdc25p (Grahl et al., 2015).

Even less is known in pathogenic fungi about the mechanisms of positive regulation of Ras protein activity through interactions with RasGEFs as no direct studies of regulation of RasGEF activity or localisation are available for these organisms. However, recent findings in the budding yeast Saccharomyces cerevisiae have revealed that the RasGEF, Cdc25p, is activated by fructose‐1,6‐bisphosate produced through glucose metabolism, linking Ras pathway activity to glycolytic flux in yeast (Peeters et al., 2017). In another model, the fission yeast *Schizosaccharomyces pombe*, two distinct RasGEFs have been shown to respond to separate signals to orchestrate Ras pathway activation during morphogenesis and mating (Papadaki, Pizon, Onken, & Chang, 2002). Together, these findings suggest that fungal RasGEFs may act to integrate multiple distinct signals for optimal cellular growth. For pathogenic fungi, the proper coordination of Ras pathway activation through RasGEFs is therefore likely important for virulence. Although little is known about how fungal RasGEFs become activated, this process is expected to be at least partially different from the model set forth by studies in higher eukaryotes. For example, in mammalian cells, RasGEF proteins are often recruited to cellular membranes and activated through a series of interactions requiring receptor tyrosine kinases (Rojas, Oliva, & Santos, 2011). The major human pathogenic fungi do not possess receptor tyrosine kinases and therefore likely have evolved different mechanisms for the control of Ras activity through RasGEF proteins.

To begin to address this knowledge gap, we explored the importance of RasGEF proteins to hyphal growth and virulence in A. fumigatus. In this study, we show that the A. fumigatus genome contains four RasGEF proteins belonging to three different RasGEF protein classes. Two of these RasGEFs (GefA and GefB) belong to the SH3‐class that is fungus‐specific. Although single deletion of each putative RasGEF was associated with only minor growth abnormalities, loss of both SH3‐class RasGEFs led to inviability of A. fumigatus in vitro and a complete loss of virulence in two models of IA. This loss of viability was underpinned by an inability to produce a polarised growth axis during germination. Further, SH3‐class RasGEF activity was required for the maintenance of polarity and hyphal structure after the initial establishment of growth. Our data suggest that, although SH3‐class RasGEFs, at least partially contribute to viability through regulation of the major Ras subfamily protein, RasA, the synthetic lethality of *gefA* and *gefB* double deletion likely involves Ras‐independent mechanisms.

## 2 RESULTS

### 2.1 A. fumigatus RasGEFs represent three classes of GEF proteins

**Figure 1:**
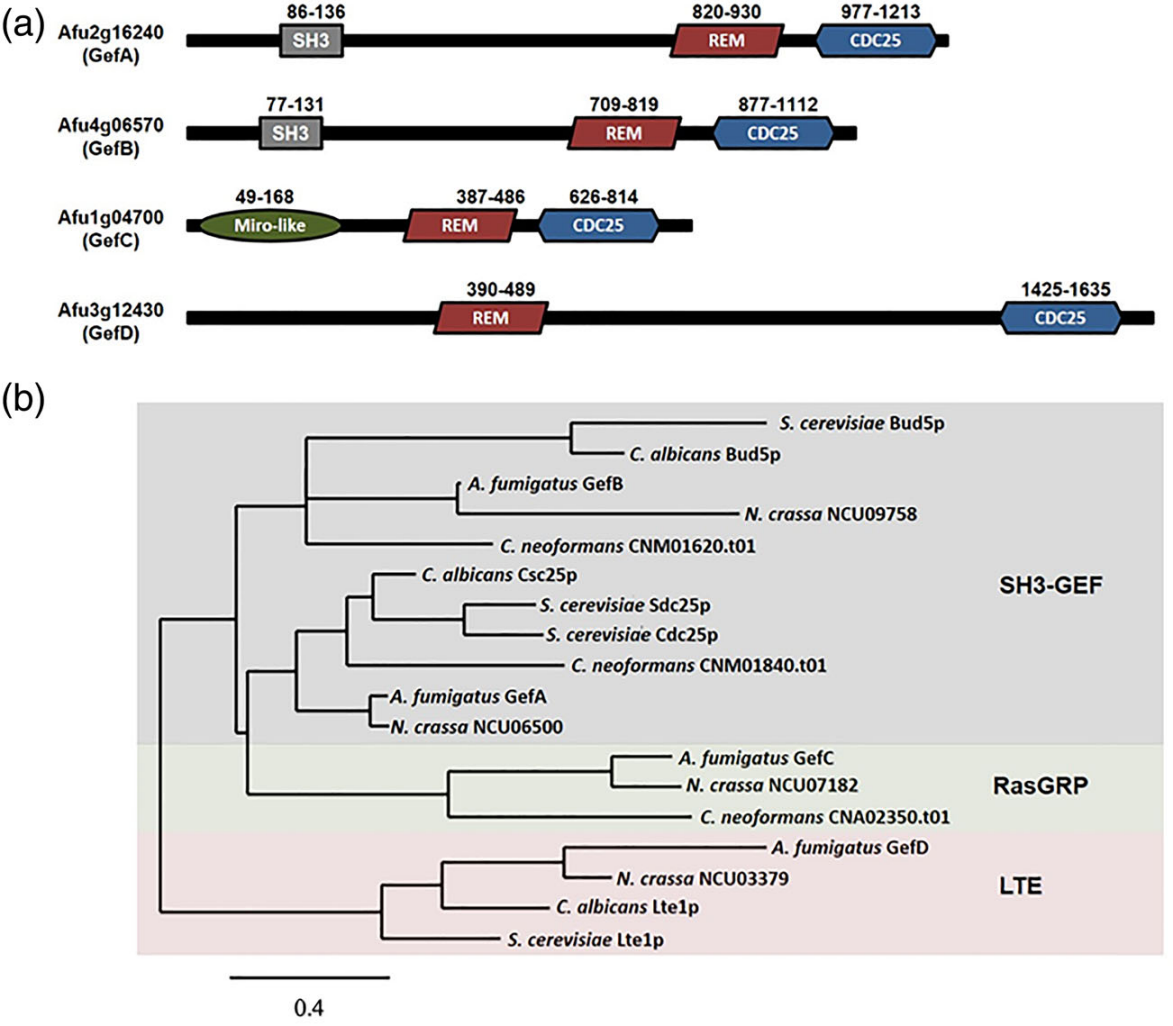
The *Aspergillus fumigatus* genome encodes four putative Ras‐subfamily‐specific guanine nucleotide exchange factors (RasGEFs) belonging to three RasGEF protein classes. (a) Basic protein domain structure of putative RasGEFs. Each RasGEF protein contains a Ras exchange motif (REM) and a CDC25 catalytic domain. GefA and GefB additionally encode a single SH3 domain at their N‐termini. GefC is predicted to encode a mitochondrial Rho‐like protein domain at the N‐terminus. (b) Phylogram of selected fungal RasGEFs including model and pathogenic fungi. Analysis was performed in the Phylogeny.fr platform and sequences were aligned with MUSCLE (v3.8.31) configured for highest accuracy. Ambiguous regions were removed with Gblocks (v0.91b). The phylogenetic tree was reconstructed using the maximum likelihood method implemented in the PhyML program (v3.1/3.0 aLRT). Graphical representation and edition of the phylogenetic tree were performed with TreeDyn (v198.3). A. fumigatus GefA and GefB cluster within the SH3‐class, whereas GefC and GefD cluster with RasGRP and LTE classes, respectively. Organisms utilised for comparison with A. fumigatus are: Saccharomyces cerevisiae, Candida albicans, Cryptococcus neoformans, and *Neurospora crassa*. Genome databases for each organism were queried with the S. cerevisiae Cdc25p sequence to identify putative RasGEF proteins for alignment. RasGEF classes were delineated based on (van Dam et al., 2009)

The A. fumigatus genome has been reported to encode four putative RasGEFs (van Dam, Rehmann, Bos, & Snel, 2009). To confirm, we performed a homology BLAST search against the A. fumigatus genome (FungiDB; Genome Version 2015‐09‐27) using the Cdc25p RasGEF sequence from S. cerevisiae. This search returned four putative protein‐encoding loci: Afu2g16240, Afu4g06570, Afu1g04700, and Afu3g12430. Each putative RasGEF protein shared an identity of 27.75%, 23.65%, 20.99%, and 21.52%, respectively, to the Cdc25p from S. cerevisiae. Each putative protein was predicted to harbour a Ras exchange motif (REM), involved in the stabilisation of RasGEF binding to Ras proteins, and a CDC25‐homology domain, which contains the catalytic region homologous to S. cerevisiae Cdc25p and is responsible for catalysing GDP release from Ras (Vigil, Cherfils, Rossman, & Der, 2010). Therefore, these four putative proteins were named GefA, GefB, GefC, and GefD, respectively (Figure 1a).

In addition to the highly conserved REM and CDC25‐homology domains, RasGEFs can be divided into different classes based on additional conserved protein domains (van Dam et al., 2009). Based on phylogenetic analyses using putative and verified RasGEF proteins from both model and pathogenic fungi, GefA and GefB belong to SH3‐class RasGEFs due to the presence of an N‐terminal SH3 domain (Figure 1a,b). This class of RasGEFs is exclusively found in fungal organisms (van Dam et al., 2009). GefA displays highest homology to the known Ras1/2p GEF from S. cerevisiae, Cdc25p, suggesting it may be the major RasA regulator in A. fumigatus. In contrast, GefB shares higher homology with Bud5p, a verified RasGEF‐controlling activation of the bud site selection‐regulator Rsr1p (Bender, 1993). GefC is a member of the RAS guanyl nucleotide‐releasing protein (RasGRP)–class RasGEFs, showing homology to predicted proteins in the model filamentous fungus, *Neurospora crassa*, and the human fungal pathogen, C. neoformans (Figure 1b). Although members of this class are found in both higher and lower eukaryotes, fungal RasGRP‐class proteins are unique in that they encode a mitochondrial Rho (Miro) domain at their N‐terminus (Figure 1a). Miro is an atypical small GTPase that, in S. cerevisiae, is a component of the ERMES complex that links the endoplasmic reticulum to mitochondria, suggesting a role in mitochondrial regulation for this RasGEF (Michel & Kornmann, 2012). Although higher eukaryotes express homologous Miro proteins, only fungal organisms express RasGEFs containing Miro domains (van Dam et al., 2009). Interestingly, GefC homologues were only identified in this study in organisms that express homologues of A. fumigatus RasB (Fortwendel, 2015; Fortwendel et al., 2005). Finally, GefD is an LTE‐class RasGEF, showing homology to Lte1p from S. cerevisiae and C. albicans in addition to a putative RasGEF protein from N. crassa (Figure 1b). S. cerevisiae Lte1p contains conserved RasGEF domains, but the presence of GEF activity in this protein is unclear (Geymonat et al., 2002). Despite a potential lack of GEF activity, Lte1p is known to be important for mitotic exit in *S*. *cerevisiae* and the homologues described here in C. albicans, N. crassa, and A. fumigatus may play similar roles.

### 2.2 RasGEF activity contributes to hyphal growth and morphogenesis

To define the importance of each RasGEF to the growth and virulence of A. fumigatus, we first generated single‐deletion strains in the uracil auxotrophic KU80Δ*pyrG* genetic background (da Silva Ferreira et al., 2006) by replacing each complete ORF with the *Aspergillus parasiticus* pyrG gene. After confirming deletion, each mutant was subsequently complemented by ectopic integration of the wild‐type locus. The strain *ΔakuB‐pyrG* ^*+*^ was used as a control strain for the assays described herein (Al Abdallah, Martin‐Vicente, Souza, Ge, & Fortwendel, 2018).

**Figure 2:**
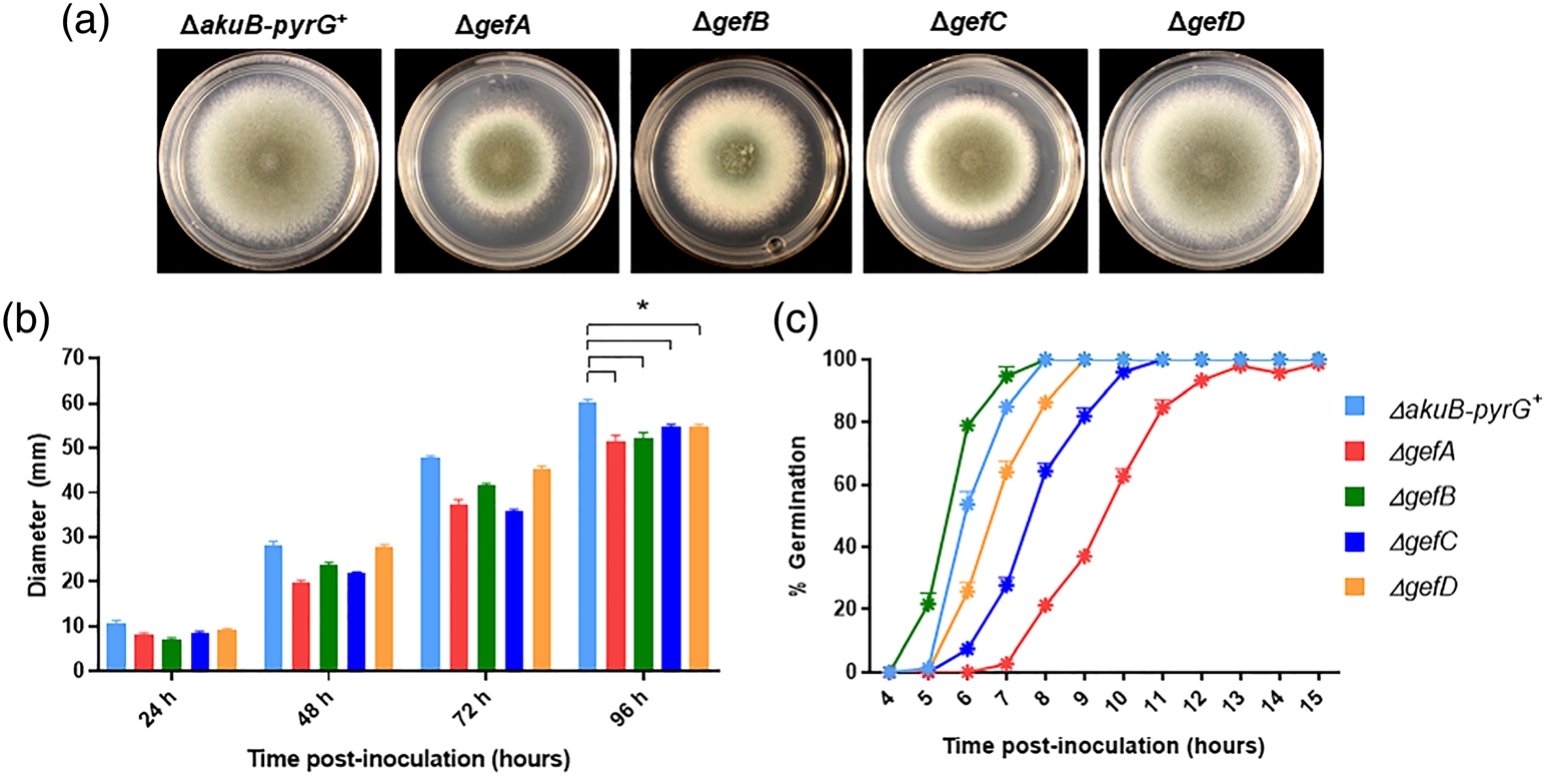
Ras‐subfamily‐specific guanine nucleotide exchange factors (RasGEFs) are required for normal *Aspergillus fumigatus* hyphal growth and germination rates. (a) Colony morphology of control and single RasGEF deletion strains. Glucose minimal medium (GMM) agar plates were inoculated with 10,000 conidia and incubated for 96 hr at 37°C. (b) Colony diameters were measured every 24 hr for the 96‐hr incubation period. Colony diameters between strains at each 24‐hr interval were compared using two‐way analysis of variance with Tukey's test for multiple comparisons (GraphPad Prism v7). Asterisks indicate a statistically significant difference (*p*‐value < 0.05) between the deletion strains and the control strain. (c) Ten thousand conidia from each strain were inoculated into GMM broth, poured over sterile coverslips and incubated at 37°C. The percentage of germination was scored as the number of conidia producing a visible germ tube amongst 100 total enumerated conidia. All experiments were performed in triplicates. Measurements and error bars represent the mean ± the standard deviation

As RasGEFs are expected to mediate the activation of RasA and loss of RasA activity leads to decreased growth and aberrant hyphal morphogenesis, the mutant strains were first characterised for alterations in macro‐ and micromorphology. Although no differences were observed in overall colony morphology, all RasGEF‐deficient strains grew at a slower rate in comparison with the control strain (Figure 2a,b). Specifically, we observed statistically significant differences in colony diameter after 72 and 96 hr of growth at 37°C for all RasGEF deletion mutants in comparison with the control strain (Figure 2b). Ectopic reintegration of each RasGEF coding region demonstrated that phenotypic changes resulted from targeted gene deletion (data not shown).

To see if the reduced colony size was associated with delayed polarity establishment, a germination assay was performed using the production of a germ tube as the assay endpoint. A delay in germination of 2 hr and 1 hr was observed for Δ*gefA* and Δ*gefC*, respectively (Figure 2c). We observed that, after 7 hr of incubation at 37°C, only 2.7% (±0.58%) and 27.7% (±2.52%) of conidia from Δ*gefA* and Δ*gefC*, respectively, had developed a germ tube, in comparison with 85% (±1%) of conidia from the control strain. Further, 100% of conidia from the control strain had germinated after 8 hr of incubation, whereas germ tubes formed in only 21.1% (±1.15%) of Δ*gefA* conidia by this time point. The Δ*gefD* mutant displayed a phenotype similar to the control strain, and, although initiation of germination was not delayed, only 25.7% (±3.06%) of the conidia had formed germ tubes after 6 hr versus 53.7% (±4.16%) of the control strain. The Δ*gefB* mutant, on the contrary, germinated faster than the control strain. After 5 hr of incubation, 21.7% (±3.51%) of conidia had developed a germ tube in comparison with 1.33% (±0.58%) of conidia from the control strain, and after 6 hr, almost 80% (±1%) of conidia from the knockout were germinated versus a 53.7% (±4.16%) from the control.

**Figure 3:**
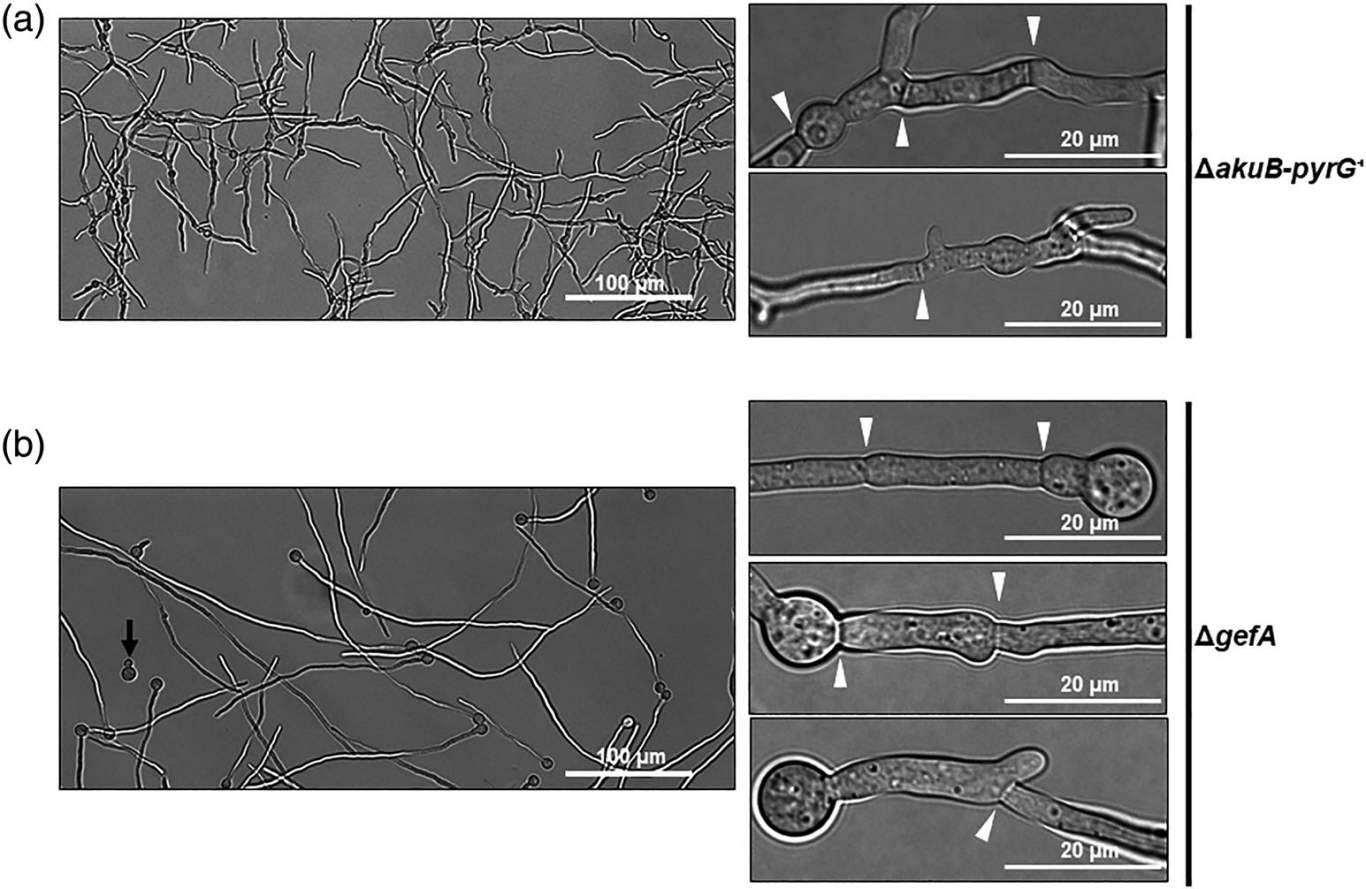
Loss of *gefA* reduces hyphal branching during early growth. Two thousand conidia from control and Δ*gefA* strains were inoculated into liquid glucose minimal medium and inoculated onto sterile coverslips. Microphotographs were taken after 16 hr at 37°C. The delay in germination previously noted for the Δ*gefA* strain was accompanied by exaggerated swelling of the germinating conidium and reduced branch emergence in the *gefA* mutant (b, left panel) versus the control strain (a, left panel). During mature hyphal growth, branch emergence in the Δ*gefA* strain is associated with swollen pre‐septal compartments (b, right panel). In contrast, the control strain was able to maintain an even hyphal volume during septation and branching (a, right panel). White arrowheads indicate septa

Although microscopic examination of germling‐phase growth revealed no aberrancies for the majority of the mutants, loss of *gefA* caused impaired branching and the formation of swollen preseptal compartments (Figure 3b). This was in contrast to the control strain that developed multi‐branched germlings with equal width preseptal and postseptal compartments (Figure 3a).

### 2.3 SH3‐class RasGEFs contribute to cell wall stability

**Figure 4:**
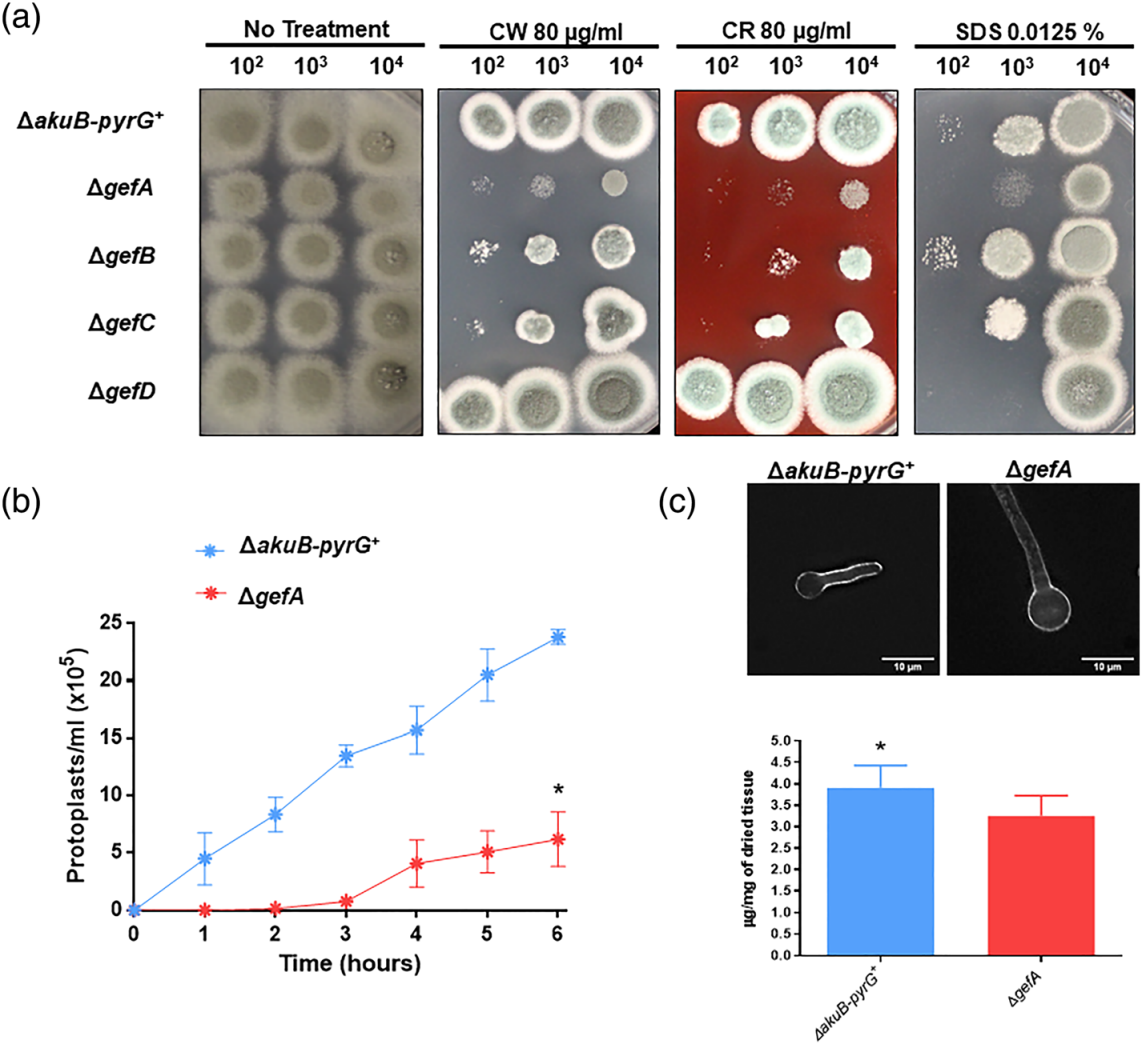
SH3‐class and RAS guanyl nucleotide‐releasing protein‐class Ras‐subfamily‐specific guanine nucleotide exchange factors are required for response to cell wall stress. (a) 100 to 10,000 conidia were point‐inoculated onto glucose minimal medium (GMM) containing 80 μg/ml of calcofluor white (CW), 80 μg/ml of congo red (CR) or 0.0125% of sodium dodecyl sulphate (SDS). Plates were incubated at 37°C for 72 hr. Addition of CW and CR to the media resulted in exaggerated growth inhibition of Δ*gefA,* Δ*gefB*, and Δ*gefC* and, in addition, SDS also inhibited growth of Δ*gefA*. (b) The Δ*gefA* cell wall is resistant to enzymatic digestion. Equal numbers of germlings from control and Δ*gefA* strains were incubated in osmotic medium containing 0.05 g/ml of a mixture of polygalacturonase and β‐glucanase at 32°C with shaking at 75 rpm. Aliquots were taken every hour over a 6‐hr time‐course and protoplasts were counted by haemocytometer at each time point. Bars indicate standard error of three independent experiments. Number of protoplasts were compared between both strains using Two‐way analysis of variance with Tukey's test for multiple comparisons (GraphPad v7). * indicates *p* < 0.05. (c) Top panels: deconvolved microphotographs of immunostained β‐glucan in nascent hyphae from the control strain and the Δ*gefA* mutant. 2 × 10^7^ conidia were grown in GMM at 37°C and incubated for 1 hr with Fc‐hDectin‐1a and another hour with an FITC‐conjugated anti‐human IgG Fc secondary antibody. Photographs were taken with a Nikon Ni‐U microscope equipped with a green fluorescent protein filter. Bottom panels: β‐glucan content analysis of the control and Δ*gefA* strains. The mutant displayed a consistent, statistically significant decrease in β‐glucan content. Data are the average of three independent experiments and represent the mean μg of β‐glucan/mg of dry weight hyphal mass (±SD). * indicates *p* < 0.05

Previous studies with yeast RasGEF and Ras mutants revealed roles in heat, oxidative, osmotic, and ionic stress resistance (Boy‐Marcotte, Ikonomi, & Jacquet, 1996; Folch‐Mallol et al., 2004). In addition, we have previously shown that Ras signalling is required for cell wall stability in A. fumigatus (Fortwendel et al., 2008; Norton, Al Abdallah, Hill, Lovingood, & Fortwendel, 2017). To determine potential involvement of A. fumigatus RasGEFs in any of the aforementioned stress responses, growth susceptibility assays interrogating cell wall, oxidative, and osmotic stresses were tested. The susceptibility of each deletion strain to cell wall stress was first examined using Calcofluor White, Congo Red, and SDS, general agents targeting the cell wall or membrane, as previously described (Al Abdallah et al., 2018). By spot dilution assay, the Δ*gefA,* Δ*gefB*, and Δ*gefC* mutants were each found to be hyper‐susceptible to cell wall disruption by Calcofluor White and Congo Red, whereas Δ*gefA*, Δ*gefD* and, to a lesser extent, Δ*gefC* displayed increased susceptibility to SDS‐induced membrane disruption (Figure 4a). When tested using the same spot dilution assay, susceptibility to osmotic stress induced by adding KCl, NaCl and sorbitol to the growth medium was unaltered as well as to oxidative stress induced by H_2_O_2_ employing a disc diffusion assay (data not shown).

To examine the RasGEF mutants for susceptibility changes to specific inhibitors of cell wall biosynthesis, we also performed broth microdilution assays using the β‐glucan synthase inhibitor, caspofungin, and the chitin synthase inhibitor, nikkomycin Z. These assays revealed an increased susceptibility to caspofungin for the Δ*gefA* strain (MEC = 0.0125 μg/ml) in comparison with the control (MEC = 0.5 μg/ml). A similar pattern was observed in the presence of nikkomycin Z, with the Δ*gefA* strain displaying an MIC‐2 (50% growth inhibitioin) of 4 μg/ml and the control strain at 16 μg/ml. The growth inhibition effects were specific to cell wall‐active antifungals, as no differences were observed when ergosterol biosynthesis was inhibited by voriconazole treatment (data not shown). As further evidence of cell wall alteration in the Δ*gefA* strain, we observed an increased resistance of this mutant to digestion by the mixture of β‐glucanase and polygalacturonase commonly used in our lab for the A. fumigatus protoplasting process. In a lysis kinetic assay, similar numbers of germlings of the Δ*gefA* and control strains were incubated with the protoplasting enzyme solution for 6 hr with aliquots taken every hour for quantification of protoplasts. Surprisingly, after 2 hr of incubation, we observed 1.25 × 10^4^ protoplasts/ml liberated from Δ*gefA* germlings in comparison with 7 × 10^5^ protoplasts/ml of the control strain. At the end of the 6‐hr experiment, the Δ*gefA* strain had generated 6.2 × 10^5^ protoplasts/ml in comparison with 2.4 × 10^6^ protoplasts/ml of the control strain (Figure 4b). These findings suggest that the alterations in responses to cell wall stress seen in the Δ*gefA* mutant may be underpinned by perturbations in the quantity or organisation of cell wall carbohydrate moieties.

To identify potential changes in the organisation or content of the cell wall upon loss of *gefA*, the control strain and Δ*gefA* mutant were subjected to β‐glucan staining and content analyses. Fluorescence microscopy revealed intense staining along the hyphal periphery of the control strain, with only a faint signal detected in the basal conidium (Figure 4c, upper panels). In contrast, the Δ*gefA* mutant stained with lower overall intensity along the hyphal cell wall (Figure 4c, upper panels). These findings suggested a potentially lower amount of β‐glucan in Δ*gefA*. To test this, we performed a β‐glucan content assay and observed a reduction of 17% in β‐glucan content in the Δ*gefA* in comparison with the control strain (Figure 4c, lower panel).

### 2.4 SH3‐class RasGEFs are essential for A. fumigatus viability

Although we previously reported that loss of A. fumigatus RasA activity leads to greatly reduced growth rates, we observed here that single deletion of RasGEFs did not have an exaggerated impact on A. fumigatus growth rate. This discrepancy implied that no single RasGEF is essential for proper activation of RasA in A. fumigatus. However, the potential exists for redundant function of these GEF proteins, especially as two of the proteins identified here, GefA and GefB, are both SH3‐class GEFs that share homology to the Cdc25p RasGEF from S. cerevisiae. Therefore, we hypothesized that these RasGEFs may redundantly function as RasA activators in A. fumigatus. In order to test this hypothesis, we first attempted to delete both SH3‐GEFs in the same strain. However, a double deletion was unobtainable after several transformation attempts, suggesting that GefA and GefB are together essential, not just for normal growth but for viability of A. fumigatus.

**Figure 5:**
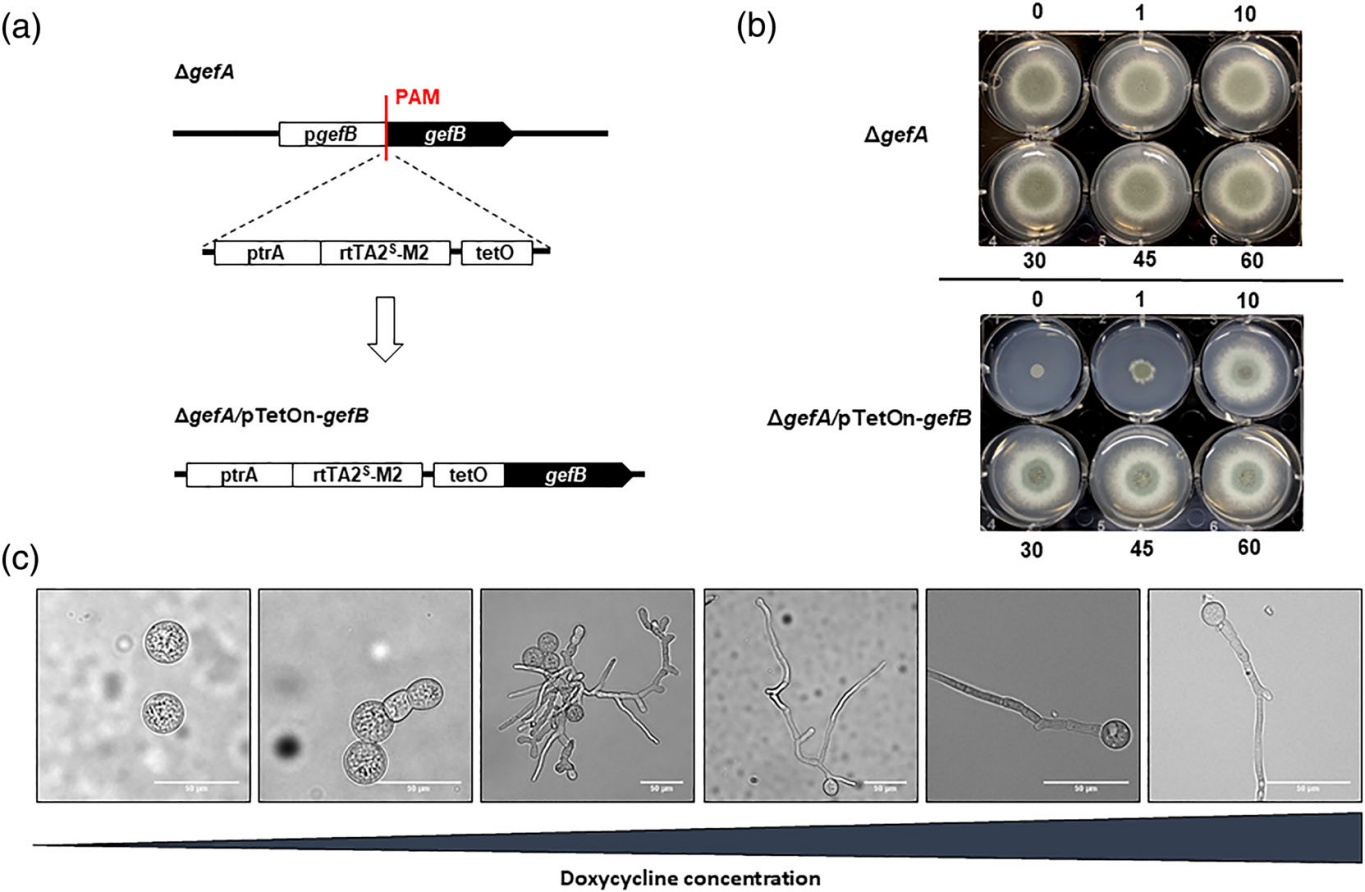
SH3‐class GEFs are essential for *Aspergillus fumigatus* viability. (a) Schematic of pTetOn promoter replacement for *gefB*. A suitable Protospacer adjacent motif site in the *gefB* promoter was targeted with a repair template carrying the pyrithiamine resistance cassette upstream of the tetracycline‐inducible (TetOn) promoter system in the Δ*gefA* genetic background to generate the Δ*gefA*/pTetOn‐*gefB* mutant. (b) Hyphal growth is responsive to exogenous addition of doxycycline. Ten thousand conidia of control and Δ*gefA*/pTetOn‐*gefB* strains were inoculated onto glucose minimal medium (GMM) agar with increasing concentrations of doxycycline. Photographs were taken after 72 hr of culture at 37°C. (c) Two thousand conidia of Δ*gefA*/pTetOn‐*gefB* were inoculated into GMM liquid media with 0, 1, 10, 30, 45, or 60 μg/ml of doxycycline (left to right) and incubated at 37°C for 24 hr. In the absence of doxycycline, the double mutant failed to generate a polarised growth axis, and was instead characterised by exaggerated swelling of conidia (left most panel). Aberrant hyphae morphologies were restored at higher concentrations of doxycycline and full recovery of the Δ*gefA* phenotype was noted at 45 μg/ml of doxycycline

To circumvent this problem, we performed a promoter mutation to replace the native promoter of *gefB* with a tetracycline‐inducible promoter (pTetOn) in the genetic background of Δ*gefA* (Figure 5a). This new strain, named Δ*gefA*/pTetOn‐*gefB*, allowed experimental study in a genetic background with only one SH3‐class RasGEF that could be up‐ or down‐regulated in the presence or absence of doxycycline, respectively (Wanka et al., 2016). Culture of Δ*gefA*/pTetOn‐*gefB* on glucose minimal medium (GMM) agar in the absence of doxycycline resulted in the development of only pinpoint colonies that were unable to form hyphae (Figure 5b). Recovery of full hyphal growth was achieved under increasing amounts of doxycycline in the medium, with maximal recovery evident at 45 μg/ml of doxycycline (Figure 5b). Microscopic examination of a similar doxycycline titration experiment in submerged culture showed that, under non‐inducing conditions, the Δ*gefA*/pTetOn‐*gefB* strain displayed an inability to produce polarised growth axes, forming only swollen conidia that were 20 times larger than those of the control strain (Figure 5c and data not shown). Addition of 1 μg/ml doxycycline resulted in the formation of wide, dysmorphic hyphal structures amongst sporadic swollen conidia (Figure 5c). Full recovery of the Δ*gefA* hyphal phenotype was evident at 45 μg/ml doxycycline, corroborating results achieved via agar‐based culture (Figure 5c). Together, these findings suggest that loss of both SH3‐class RasGEFs produces lethality via loss of polarity establishment during early growth.

**Figure 6:**
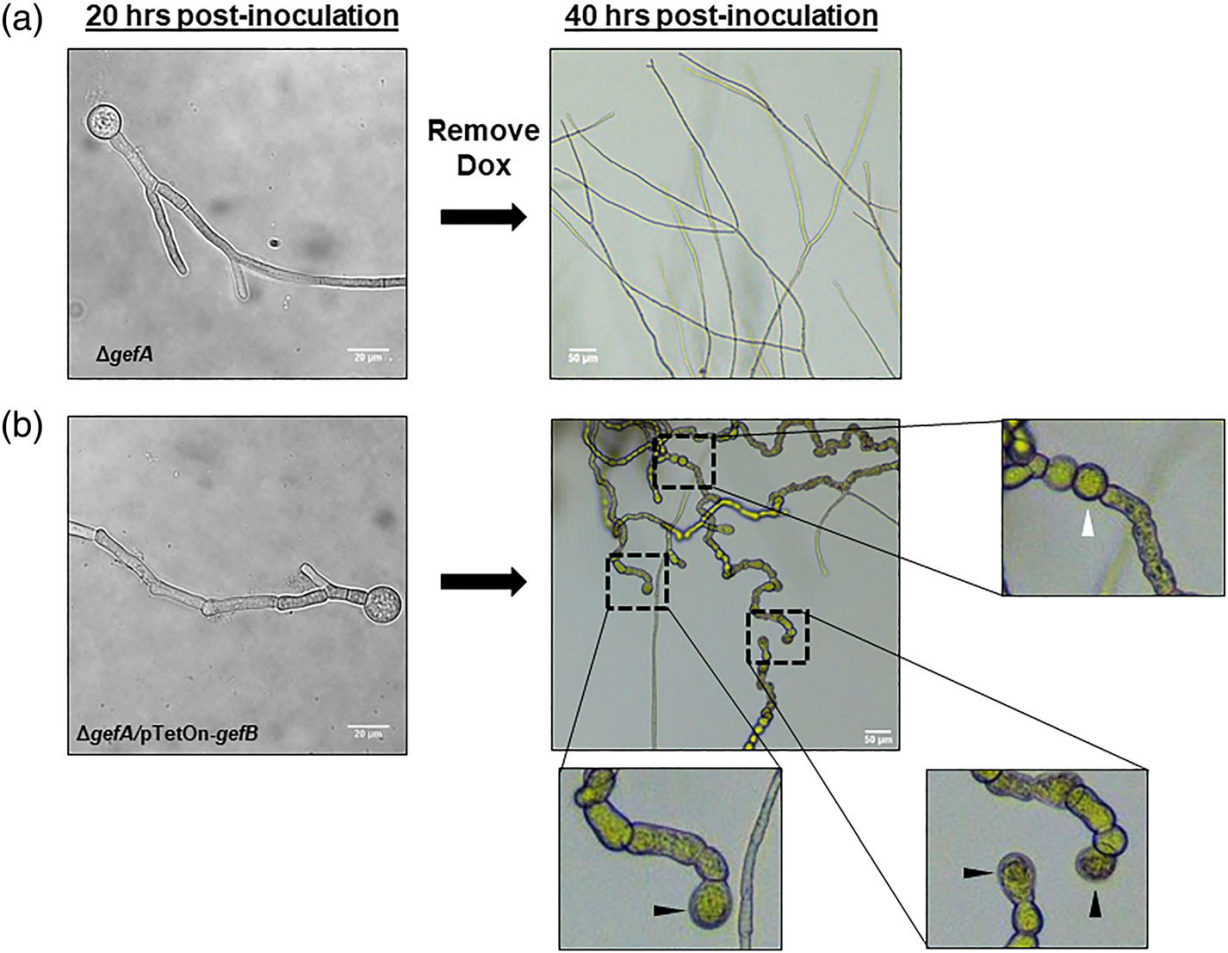
SH3‐class Ras‐subfamily‐specific guanine nucleotide exchange factors (RasGEFs) are required for maintenance of hyphal structure postpolarity establishment. Two thousand conidia of Δ*gefA* (A) and Δ*gefA*/pTetOn‐*gefB* (b) were inoculated in glucose minimal medium (GMM) containing 30 μg/ml of doxycycline and incubated at 37°C for 20 hr. After this time, the culture medium was replaced by fresh GMM without doxycycline and the strains were incubated for 20 additional hours. After 20 hr in the presence of doxycycline, the Δ*gefA*/pTetOn‐*gefB* was indistinguishable from the parent strain (left panels). However, after further incubation in doxycycline‐free media (right panels), the Δ*gefA*/pTetOn‐*gefB* mutant developed swollen basal compartments (denoted by a white arrowhead in the enlarged panel) as well as swollen hyphal tips (denoted by black arrowheads in enlarged panels) and subapical compartments, suggesting a loss of polarised morphogenesis

We next questioned the importance of SH3‐class RasGEF activity for normal growth after the establishment of polarity. To do this, Δ*gefA*/pTetOn‐*gefB* and the Δ*gefA* parent strain were incubated overnight in liquid media containing doxycycline. This ensured Δ*gefB* expression during early growth and resulted in the development of Δ*gefA*/pTetOn‐*gefB* germlings that resembled those of the parent strain (Figure 6a,b). After this early growth phase, the doxycycline‐containing media was replaced with fresh culture media containing no doxycycline to halt induction of Δ*gefB* expression. Following 20 more hours of culture, the Δ*gefA*/pTetOn‐*gefB* strain developed swollen hyphal tips, indicating an induced loss of polarity (Figure 6b). This result correlated with our earlier data delineating a requirement for SH3‐class RasGEFs for establishment of polarised growth. However, the subapical compartments of the Δ*gefA*/pTetOn‐*gefB* strain also became swollen and dysmorphic, indicating a loss of hyphal structural maintenance even in nonapical regions (Figure 6b). Taken together, our data indicate roles for SH3‐class RasGEFs not only in establishing hyphal growth, but also in the maintenance of hyphal structure.

**Figure 7:**
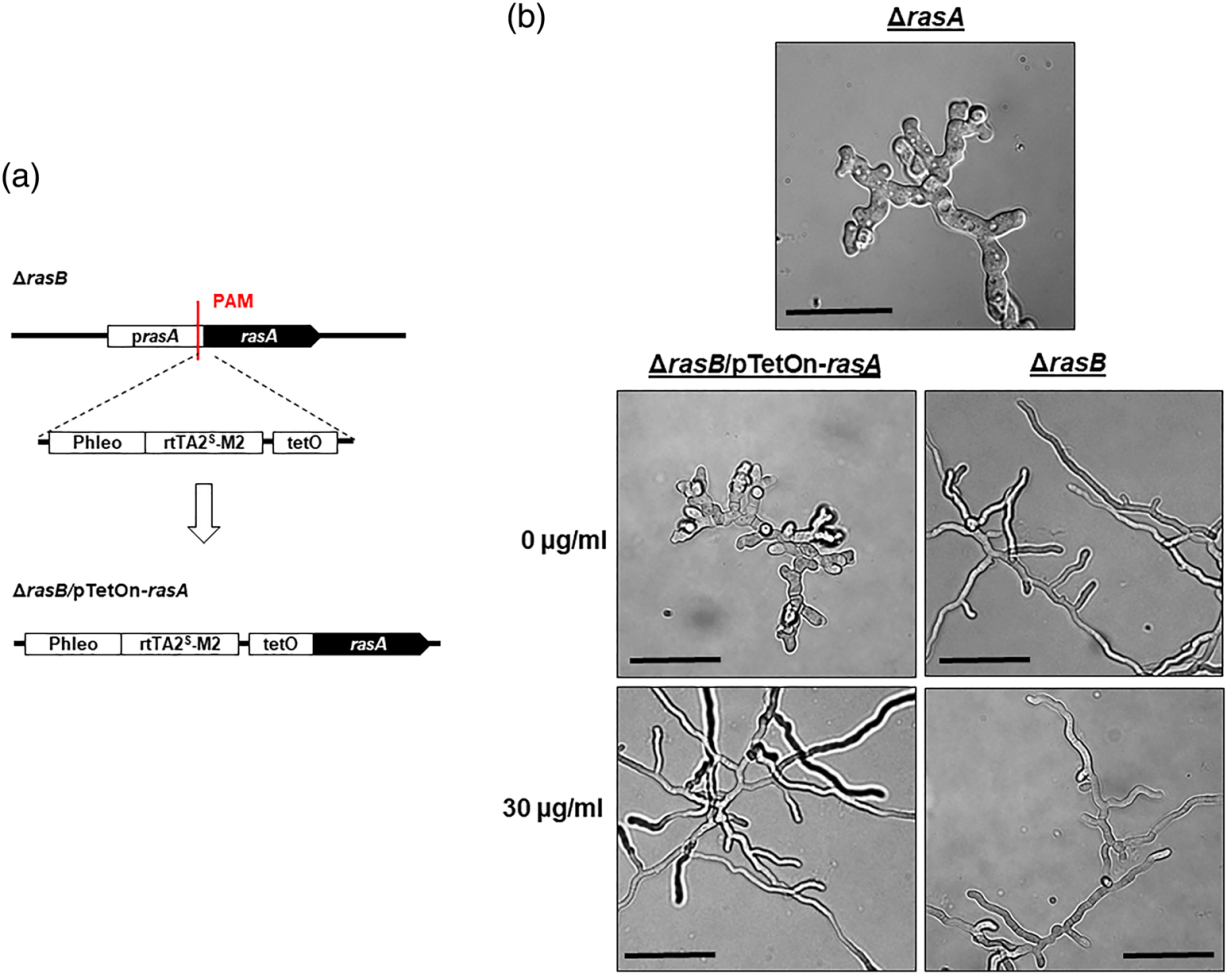
Loss of both Ras subfamily genes, *rasA* and *rasB*, does not phenocopy the non‐induced Δ*gefA*/pTetOn‐*gefB* strain. (A) Schematic of pTetOn promoter replacement for *rasA*. Similar to the technique used for *gefB*, a suitable protospacer adjacent motif site in the *rasA* promoter was targeted with a repair template carrying the pyrithiamine resistance cassette upstream of the tetracycline‐inducible (TetOn) promoter system in the Δ*rasB* genetic background to generate the Δ*rasB*/pTetOn‐*rasA* mutant. (b) Loss of both *rasA* and *rasB* does not exaggerate the Δ*rasA* hyphal phenotype. Two thousand conidia of the Δ*rasA*, Δ*rasB* and Δ*rasB*/pTetOn‐*rasA* strains were inoculated into glucose minimal medium (GMM) liquid media with 0 or 30 μg/ml of doxycycline and incubated at 37°C for 24 hr. In the absence of doxycycline (middle panels), the double mutant formed stunted, highly branched hyphae similar to previously reported phenotypes of the Δ*rasA* strain (top panel). Aberrant hyphal morphologies were restored at 30 μg/ml doxycycline (bottom panels) and the Δ*rasB* mutant grew unaffected by the presence of doxycycline. Scale bar, 25 μm

We next hypothesized that, because A. fumigatus expresses two Ras subfamily homologues, the synthetic lethality resulting from mutation of both SH3‐class RasGEFs may result from simultaneous deactivation of RasA and RasB. To test this, we performed a mutation similar to the earlier *gefB* promoter replacement, using our CRISPR/Cas9 system to target a protospacer adjacent motif site upstream of the *rasA* coding sequence in a Δ*rasB* genetic background (Figure 7a). Growth of the resulting strain, Δ*rasB*/pTetOn‐*rasA*, was examined in the presence and absence of doxycycline and compared with the Δ*rasB* parent. Hyphae of the Δ*rasB* mutant were previously described as slow growing but morphologically similar to wild type (Fortwendel et al., 2005), phenotypes that were unaltered by the presence of doxycycline in our studies (Figure 7b). At 0 μg/ml doxycycline, the double mutant formed stunted, highly branched hyphae that exactly phenocopied the previously reported Δ*rasA* mutant, a strain that has a clear polarity defect with stunted, hyper‐branched hyphae (Figure 7b and Fortwendel et al., 2008). These data indicate that loss of both *rasA* and *rasB* (Δ*rasB*/pTetOn‐*rasA* cultured with no doxycycline) did not cause an exacerbation of the Δ*rasA* single mutant phenotype (Figure 7b and Fortwendel et al., 2008). In contrast, growth in the presence of 30 μg/ml doxycycline, to induce *rasA* expression, resulted in a strain that mimicked the Δ*rasB* single mutant (Figure 7b). Taken together, these findings suggested that GefA and GefB likely orchestrate polarity establishment through RasA and additional RasA/B‐independent mechanisms.

### 2.5 RasA protein abundance is dependent on SH3‐class RasGEF activity

**Figure 8:**
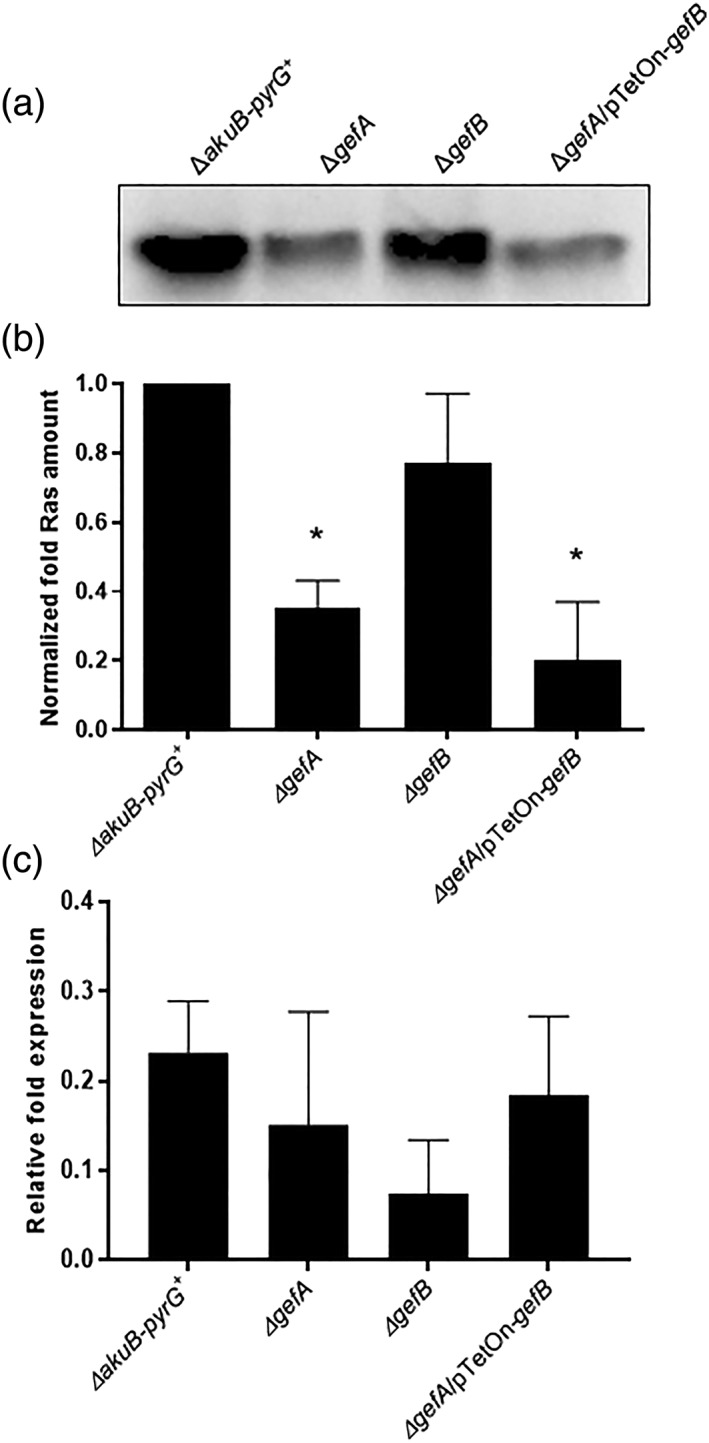
GefA is important for maintenance of RasA protein abundance. (a) Representative immunoblot comparing steady‐state RasA protein levels amongst the control, Δ*gefA*, Δ*gefB*, and Δ*gefA*/pTetOn‐*gefB* strains employing lysates extracted from cultures grown under non‐inducing conditions (no doxycycline). The anti‐Ras, clone Ras10 used for western blot analysis is a mouse monoclonal antibody that recognises p21H‐, K‐ and N‐Ras (Millipore). Only a single band of 21 kDa, corresponding to RasA, is observed in the western blot. This band is not present in a *rasA* deletion mutant (not shown), suggesting only the RasA is recognised by the Ras10 antibody. (b) Quantitative densitometric analysis of immunoblots indicates that RasA abundance is significantly decreased by ~70% in the Δ*gefA* mutant and by ~80% in the Δ*gefA*/pTetOn‐*gefB* with respect to the control strain. Measurements and error bars represent the mean of three independent experiments. Statistical analyses were performed by using one‐way analysis of variance (ANOVA) with Dunnett's test for multiple comparisons (*, *p* < 0.05). (c) Transcript levels of *rasA* do not correlate with changes in RasA protein abundance. No statistical differences were observed in *rasA* expression levels amongst the mutant strains in comparison with the control strain. qPCR was performed using *Aspergillus fumigatus* tubulin (*tubA*) as the endogenous standard. Each sample was analysed in technical and biological triplicate. Statistics were computed by two‐way ANOVA with Tukey's test for multiple comparisons and no significant differences were observed

Although RasA/B‐independent mechanisms are likely at play, we hypothesized that either GefA or GefB must play a role in regulation of RasA. Interestingly, western blot analyses of total cellular lysates using an anti‐Ras antibody suggested that RasA protein stability is affected by the presence of GefA. Specifically, we observed a significant decrease (~70%) in Ras protein abundance in the Δ*gefA* strain when compared with the control strain (Figure 8a,b). Although the Δ*gefB* strain displayed no significant reduction in Ras protein abundance, inhibition of both SH3‐GEFs via culture of the Δ*gefA*/pTetOn‐*gefB* mutant in the absence of doxycycline resulted in an 80% reduction of Ras protein abundance (Figure 8a,b). These results suggested that the presence of GefA is required for either stabilisation of the RasA protein or that signalling cascades regulated by GefA may contribute to *rasA* gene expression via a feedback loop. To investigate this, we determined *rasA* mRNA levels by quantitative polymerase chain reaction (qPCR) under the culture conditions used for western blot analyses and observed no significant difference in *rasA* expression in the mutant strains when compared with the control strain (Figure 8c). This finding suggested that loss of GefA causes increased degradation of RasA at the protein level, not loss of gene expression.

**Figure 9:**
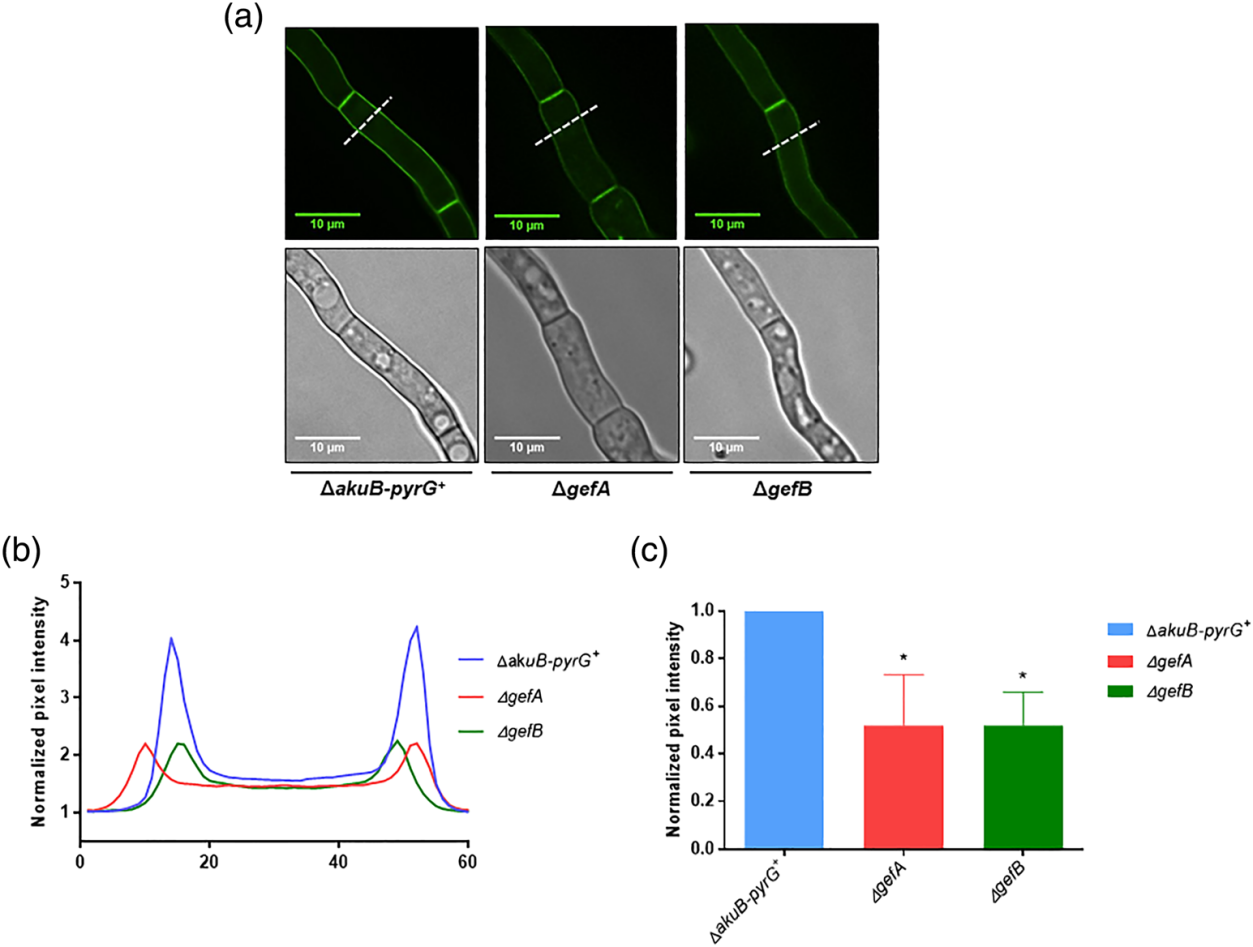
Reduction in RasA protein abundance induced by *gefA* deletion is not associated with mislocalization of RasA from the plasma membrane. (a) Single deletion of either SH3‐class Ras‐subfamily‐specific guanine nucleotide exchange factor (RasGEF) does not cause mislocalization of RasA. A green fluorescent protein (GFP)–RasA fusion protein was expressed from the endogenous *rasA* locus in the Δ*gefA* and Δ*gefB* genetic backgrounds. Deconvolved microphotographs acquired after overnight culture revealed GFP‐RasA to be localised at the hyphal periphery and septa in the control strain, as previously described, and this subcellular distribution was unchanged in the RasGEF deletion strains. (b and c) GFP signal intensity is primarily confined to the hyphal periphery, but reduced by ~50% in the Δ*gefA* and Δ*gefB* mutants. Pixel intensity, normalised to the background signal, is shown for an average of 20 randomly chosen measurements as (b) hyphal cross‐sections and (c) as peak pixel intensity for each strain. Pixel intensity was quantitated using the Nikon Advanced Research software package. Measurements and error bars represent the mean and standard deviation. Statistics were computed by one‐way analysis of variance with Dunnett's test for multiple comparison (GraphPad Prism v7). Asterisks indicate a statistically significant difference (*p*‐value < 0.05) between the deletion strains and the control strain

To see if loss of GefA was associated with RasA mislocalisation, and therefore subsequent degradation, RasA was tagged with green fluorescent protein (GFP) at the N‐terminus in the control strain and Δ*gefA* and Δ*gefB* mutants. Fluorescence microscopy analyses revealed RasA in the control strain to be localised to the plasma membrane at the hyphal periphery and septa (Figure 9a), as previously described (Fortwendel et al., 2012). RasA localisation patterns in the Δ*gefA* and Δ*gefB* mutants were identical to the control strain, indicating that loss of RasGEF activity does not affect Ras protein localisation (Figure 9a). However, the decrease in RasA protein abundance induced by deletion of *gefA* that we previously noted by western blot analyses was also evident by fluorescence microscopy in strains expressing GFP‐RasA. Pixel intensity analysis of hyphal cross sections confirmed a reduction in RasA membrane presence of ~50% in the Δ*gefA* and Δ*gefB* mutants (Figure 9b,c). Together, these data suggest that loss of GefA results in decreased RasA abundance via protein degradation.

### 2.6 SH3‐class RasGEFs are essential for growth in vivo

**Figure 10:**
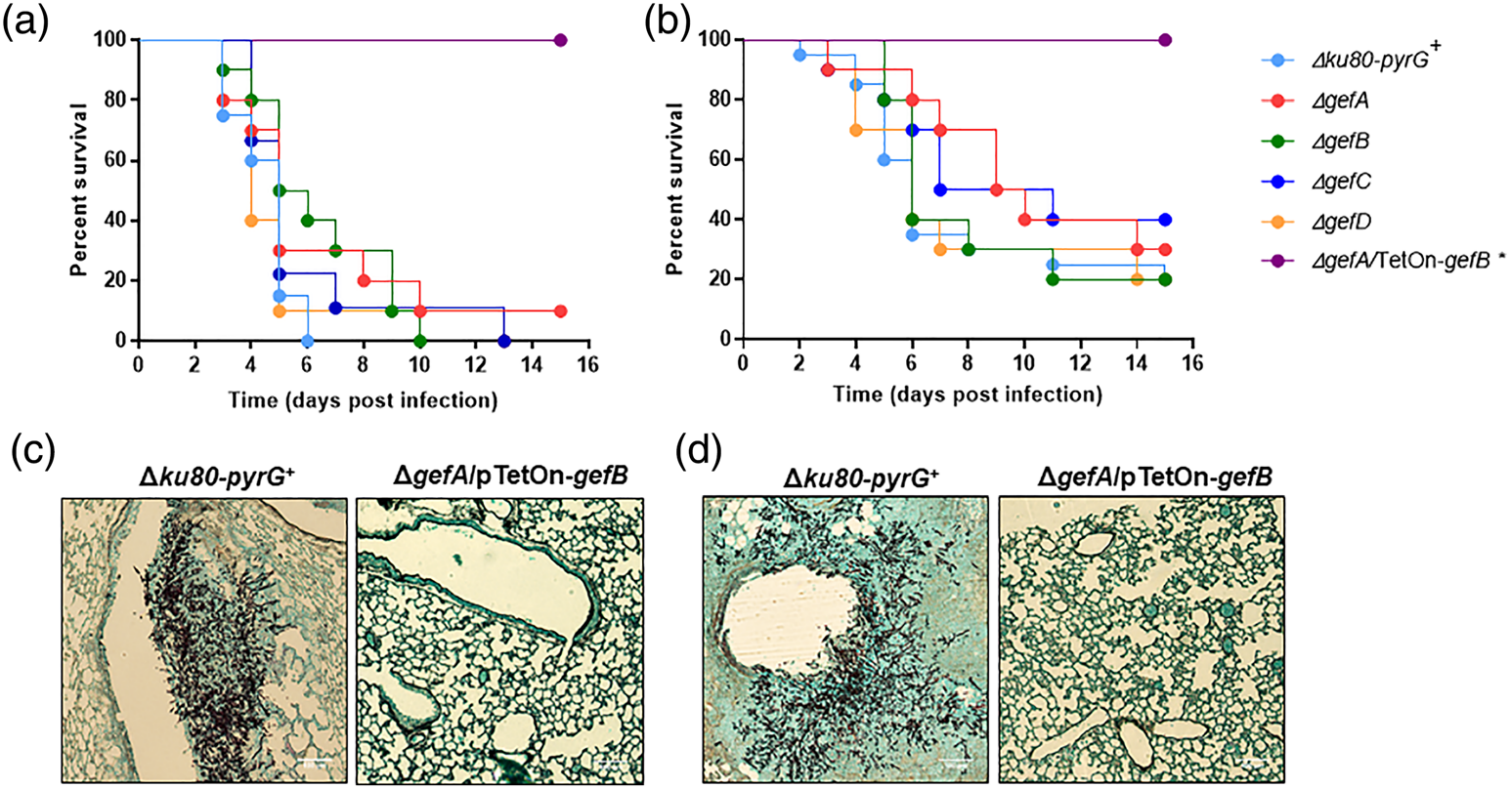
The Δ*gefA/*pTetOn*‐gefB* mutant is avirulent in two murine models of invasive pulmonary aspergillosis. Survival curves for neutropenic (a) and non‐neutropenic (b) invasive aspergillosis models. Kruskal–Wallis analysis with Dunn's test for multiple comparisons was used to compare survival curves between the mutants and the control strain. (* indicates *p* < 0,05). (c and d) Micrographs of Grocott's methenamine silver stain sections of lung tissue from mice challenged with 1 × 10^5^ conidia of the control strain and Δ*gefA*/pTetOn‐*gefB* and immunosuppressed with 150 mg/kg of cyclophosphamide and (c) 40 mg/kg of triamcinolone acetate (TA) or (d) only with TA. Microphotographs from the lungs of the mice infected with the control strain confirmed tissue invasion by identification of multiple fungal lesions. Note the absence of fungal elements in the lungs of those mice challenged with the Δ*gefA/*pTetOn*‐gefB* strain

Prolonged use of corticosteroids and other immunosuppressive agents are important risk factors for development of IA (Patterson et al., 2016). To delineate the contribution of RasGEFs to virulence, we performed two murine models of invasive pulmonary aspergillosis with different immunosuppressive regimens. For the neutropenic model, mice were treated with cyclophosphamide, an alkylating drug that causes profound neutropenia as well as with triamcinolone acetate, a corticosteroid that affects alveolar macrophages function, then reducing the first barrier to pulmonary infection. This model mimics the infection in profoundly neutropenic patients, like those undergoing leukaemia (Desoubeaux & Cray, 2017). In a separate experiment, a non‐neutropenic model, consisting only of corticosteroid administration, was employed. In both models, mice were inoculated on Day 0 and survival was followed for 15 days. Experimental arms inoculated with the RasGEF single‐deletion mutants displayed no statistically significant change in 15‐day survival rates, when compared with the control strain (Figure 10a,b). However, the Δ*gefA*/pTetOn‐*gefB* double mutant displayed complete avirulence in both models. To mimic a scenario where A. fumigatus must initiate and sustain infectious growth with no SH3‐class RasGEF activity, the Δ*gefA*/pTetOn‐*gefB* strain was cultured in vitro in the presence of doxycycline to produce conidia for inoculation, but infection was carried out in the absence of doxycycline. Whereas infection with the control strain resulted in 100% and 80% mortality in the neutropenic and non‐neutropenic model, respectively, 100% of mice challenged with Δ*gefA*/pTetOn‐*gefB* survived to day 15 (Figure 10a,b). This finding shows that the inviability produced in vitro by loss of SH3‐class RasGEF activity replicated during in vivo growth conditions.

Histopathological analysis of lung sections revealed that the virulence of the control strain in each model was associated with the development of large fungal lesions that were present mainly around major airway passages (Figure 10c,d). Hyphae of the control strain invaded both the bronchial and alveolar spaces, disrupting normal pulmonary structure and resulting in immune cell infiltration and tissue necrosis (Figure 10c,d). Similar fungal affectation was observed in the lungs from those mice infected with the single RasGEF‐deletion mutants in both models (data not shown). In contrast, fungal elements were not observed in the lungs infected with the Δ*gefA*/pTetOn‐*gefB* double mutant, confirming lack of in vivo growth with this strain (Figure 10c,d).

## 3 DISCUSSION

In the present work, we demonstrated that SH3‐class RasGEFs are essential for polarity establishment during early growth in vitro as well as for maintenance of hyphal morphogenesis after polarity has been established. The loss of polarity induced by lack of SH3‐class RasGEF activity is associated with decreased RasA protein abundance that is independent of *rasA* gene expression or of RasA protein localisation. Although steady state activity levels of RasA are expected to decrease when RasGEF activity is lost, our data are the first to describe decreased RasA protein abundance under these conditions in fungi. As GEF proteins are typically required for Ras‐protein activation, our findings suggest that inactive Ras proteins may be targeted for degradation in A. fumigatus and therefore may reveal a previously unknown regulatory mechanism modulating Ras protein stability in fungi. Alternatively, the decreased levels of RasA in the Δ*gefA* mutant may be independent of activity and instead may be due to loss of stabilising protein partners that are also a target of GefA. Finally, we also showed that SH3‐class RasGEFs are also required for A. fumigatus in vivo growth, as the Δ*gefA*/pTetOn‐*gefB* mutant was avirulent in two separate murine models of IA.

The finding that the SH3‐class RasGEFs function together to control an essential cellular process was surprising. In S. cerevisiae, the main RasGEF controlling Ras1/2p protein activity, Cdc25p, was originally reported to be an essential gene (Lai, Boguski, Broek, & Powers, 1993; Munder, Mink, & Küntzel, 1988). However, subsequent studies demonstrated that this gene is dispensable in genetic backgrounds that encode a functional Cdc25p paralog, Sdc25p (Folch‐Mallol et al., 2004). In this genetic background, the deletion of *CDC25* actually produces resistance to heat, ionic, osmotic, and oxidative stresses (Boy‐Marcotte et al., 1996; Folch‐Mallol et al., 2004). In C. albicans, the only human pathogenic fungus where a RasGEF has been partially characterised, the deletion or down‐regulation of *CDC25* is not essential but results in cells unable to filament, a known virulence‐associated attribute for this pathogen (Grahl et al., 2015; Roemer et al., 2003). As single deletion of any RasGEF did not result in a Δ*rasA* mutant phenotype, we suspected that multiple RasGEFs likely share an overlapping function in activation of RasA and that, because they are most similar to Cdc25p, the SH3‐class RasGEFs played these roles. However, rather than merely mimicking a *rasA*‐deletion phenotype, loss of both SH3‐class RasGEFs generated a germinating conidium that was unable to produce a polarised growth axis. A major difference in Ras networks between yeast organisms, like S. cerevisiae or C. albicans, and A. fumigatus is that filamentous organisms encode two distinct Ras subfamily members (Fortwendel, 2015). In A. fumigatus, these proteins are called RasA and RasB and appear to be functionally unique when compared with S. cerevisiae Ras1p and Ras2p. For example, RasA and RasB have different post‐translational modification signals, implying differing subcellular localisations, and RasB also contain protein domains distinct from those found in RasA (Fortwendel, 2015). These differences are not found when comparing S. cerevisiae Ras1p with Ras2p. Therefore, we hypothesized that RasA and RasB may have largely distinct roles and that the essential nature of SH3‐class RasGEFs in A. fumigatus may be underpinned by coregulation of both Ras subfamily proteins. However, loss of both *rasA* and *rasB* did not phenocopy *gefA* and *gefB* double mutation. Therefore, the synthetic lethality of *gefA* and *gefB* codeletion is likely dependent on proteins outside of the RasA/B subfamily.

Although S. cerevisiae and C. albicans do not encode a RasB homologue, these yeast organisms do express a Ras‐like protein called Rsr1p. Rsr1p acts as an internal polarity landmark by regulating polarisation of a Rho‐type GTPase, Cdc42p (Kang, Béven, Hariharan, & Park, 2010). In turn, this Rsr1‐Cdc42 circuit orchestrates bud‐site selection during yeast growth and, in C. albicans, tropic growth of hyphal forms (Pulver et al., 2013). Using the S. cerevisiae Rsr1p sequence to query the A. fumigatus genome database, we identified a single, uncharacterized Rsr1 homologue (Afu5g08950). The importance of Rsr1 homologues has only been studied in one filamentous fungal organism, *Ashbya gossypii*, where it regulates normal hyphal growth via localisation of required polarisome components (Bauer, Knechtle, Wendland, Helfer, & Philippsen, 2004). The known GEF for Rsr1p in S. cerevisiae is Bud5p (Bender, 1993), an SH3‐class RasGEF that, according to our phylogenetic analysis, is similar to A. fumigatus GefB (Figure 1). As expected, deletion of *BUD5* results in phenotypes mimicking loss of *RSR1* (Kang, Lee, & Park, 2004). Therefore, it is possible that GefA and/or GefB may play overlapping roles in A. fumigatus Rsr1 regulation, rather than RasB, therefore supporting the hypothesis that coordinated activation of RasA and Rsr1 are absolutely required for polarity establishment in A. fumigatus. As our Δ*gefA* and Δ*gefB* strains exhibited delayed and premature germination rates, respectively, it is likely that the coordinated action of RasA and Rsr1 pathways is important for the proper timing of polarity establishment in A. fumigatus. As GefC was only identified in our study in fungal organisms that express RasB homologues, we hypothesize that GefC homologues may be the cognate GEFs for these unique Ras subfamily proteins. More in‐depth analysis of Ras‐RasGEF interactions will be required to fully delineate Ras pathway control by specific RasGEFs.

In conclusion, the present study identifies RasGEFs important for growth and virulence in A. fumigatus. In addition, our data suggest the fungus‐specific SH3‐class RasGEFs coordinately regulate RasA/B‐dependent and ‐independent pathways to initiate polarity establishment and to maintain hyphal structure during invasive growth. Our finding that the SH3‐class RasGEFs are together essential implies that chemical inhibition of their activity could be a successful path to novel antifungal therapeutics. Although GEF‐effector protein interactions were once considered undruggable, a recent report coupling virtual and experimental screening led to the discovery of two compounds with anti‐GEF activity in mouse fibroblast cell lines (Evelyn et al., 2014). In‐depth study into the fungus‐specific nature of the SH3‐class RasGEFs will be required to pursue the possibility of selective inhibition.

## 4 EXPERIMENTAL PROCEDURES

### 4.1 Strains and growth conditions

All strains used in this study are listed in Table 1. The uridine/uracil auxotrophic A. fumigatus strain KU80Δ*pyrG* was used as the genetic background for generation of each single RasGEF deletion (da Silva Ferreira et al., 2006). For phenotypic comparisons, the strain Δ*akuB‐pyrG* ^*+*^ was generated by integrating the *pyrG* gene of *A*. *parasiticus* into the endogenous, non‐functional *pyrG* locus of the KU80Δ*pyrG* strain (Al Abdallah et al., 2018). All strains were cultured on GMM agar plates supplemented with uridine/uracil, as necessary (Shimizu & Keller, 2001). GMM broth was used for submerged cultures, unless otherwise noted. GMM supplemented with 0.5% yeast extract (GMM + YE) was used in order to potentiate growth for protein extraction.

**Table 1:** Strains used in this study

Strain	Genetic background	Source
KU80Δ*pyrG*	CEA17	Fungal Genetics Stock Center (da Silva Ferreira et al., 2006)
Δ*akuB‐pyrG* ^*+*^	KU80Δ*pyrG*	Al Abdallah et al. (2018)
Δ*gefA*	KU80Δ*pyrG*	This study
Δ*gefB*	KU80Δ*pyrG*	This study
Δ*gefC*	KU80Δ*pyrG*	This study
Δ*gefD*	KU80Δ*pyrG*	This study
Δ*gefA/*pTetOn*‐gefB*	Δ*gefA*	This study
GFP‐*rasA*	Δ*akuB‐pyrG* ^*+*^	This study
GFP‐*rasA*/Δ*gefA*	Δ*gefA*	This study
GFP‐*rasA*/Δ*gefB*	Δ*gefB*	This study
*ΔrasA*	H237	(Fortwendel et al., 2008)
*ΔrasB*	H237	(Fortwendel et al., 2005)
*ΔrasB/*pTetOn*‐rasA*	Δ*rasB*	This study
Δ*gefA::gefA*	Δ*gefA*	This study
Δ*gefB::gefB*	Δ*gefB*	This study
Δ*gefC::gefC*	Δ*gefC*	This study
Δ*gefD::gefD*	Δ*gefD*	This study

*Note*. GFP: green fluorescent protein.

For quantification of radial growth rates, 5 μl containing 10^4^ conidia suspended in sterile water were inoculated onto the centre of GMM agar plates. The colony diameter of each strain was measured daily over the first 4 days of growth at 37°C, and images were taken at the end of day four. For microscopic analyses, 10^3^ conidia were inoculated in 5 ml of GMM and poured over sterile coverslips in a sterile petri dish. These cultures were incubated for 16–24 hr at 37°C before analysis using bright field microscopy. Germination assays were carried out as previously described (Fortwendel, Panepinto, Seitz, Askew, & Rhodes, 2004). All experiments were performed in triplicate and data is provided as mean ± standard deviation.

### 4.2 Stress response and antifungal activity assays

To determine the role of the RasGEF genes in response to cell wall and osmotic stresses, 5 μl drops containing suspensions of fresh conidia were point‐inoculated onto GMM supplemented with calcofluor white (50 and 80 μg/ml), congo red (40 and 80 μg/ml), sodium dodecyl sulphate (SDS; 0.0125%), 1 M NaCl, 1 M KCl, or 1.2 M sorbitol. Colony development was monitored every 24 hr during 72 hr of incubation at 37°C. Susceptibility to oxidative stress by hydrogen peroxide (1 mM and 3 mM) was determined as previously described (Sugareva et al., 2006). The inhibition zone diameters were measured after 48 hr of incubation at 37°C. Susceptibility to caspofungin and voriconazole was determined in Roswell Park Memorial Institute medium, following the recommendations of the document M38‐A2 of Clinical and Laboratory Standards Institute (Clinical and Laboratory Standards Institute, 2008). Minimum effective concentration was determined after 24 hr and corresponded to the lowest concentration of caspofungin leading to the formation of small, rounded colonies. Minimum inhibitory concentration (MIC) of voriconazole was determined after 48 hr at 37°C and corresponded to the minimum concentration that allowed to a 100% of growth inhibition. The same methodology was used for the determination of susceptibility to the chitin synthase inhibitor, nikkomycin Z, with 50 and 100% reduction of growth (MIC‐2 and MIC‐0, respectively) after 48 hr considered as endpoints.

β‐glucanase lysis kinetic assays were performed by inoculating 1.2 × 10^8^ conidia into 100 ml of yeast glucose medium (5 g/L yeast extract and 20 g/L D‐glucose) and incubating at 32°C for 16 hr with agitation at 250 rpm. The resulting hyphal mat was filtered and washed twice with sterile deionised water and the germlings were incubated in osmotic medium (1.2 M MgSO_4_ × 7H_2_O, 10 mM sodium phosphate buffer) containing 0.05 g/ml of a mixture of polygalacturonase and β‐glucanase enzymes (VinoTaste®, Novozymes, Bagsværd, Denmark) at 32°C and 75 rpm. Aliquots were taken every hour over a 6‐hr time‐course and protoplasts were counted by haemocytometer at each time point.

All analyses were performed in triplicate and data are presented as the mean ± standard deviation.

### 4.3 Generation of mutant strains

To confirm previously reported putative RasGEF proteins encoded by the A. fumigatus genome (van Dam et al., 2009), a BLAST search was performed using the S. cerevisiae Cdc25p protein sequence. Four genes were found to encode for RasGEF homologues in A. fumigatus, each containing conserved RasGEF protein domains. These genes, identified by systematic name, were Afu2g16240, Afu4g06570, Afu1g04700, and Afu3g12430 and were named *gefA*, *gefB*, *gefC* and *gefD*, respectively. Single‐gene deletion mutant strains were generated in the A. fumigatus KU80Δ*pyrG* genetic background (da Silva Ferreira et al., 2006). This strain is deficient in orotidine 5′‐monophosphate decarboxylase activity and, therefore, auxotrophic for uridine or uracil. For the generation of each single‐gene knockout, the *A*. *parasiticus* pyrG cassette (Calvo, Bok, Brooks, & Keller, 2004) was used to completely replace each RasGEF ORF, generating a complete gene deletion. Protoplast generation and fungal transformations were performed following previously described protocols (Yelton, Hamer, & Timberlake, 1984). Transformants were genotyped by polymerase chain reaction (PCR) of genomic DNA and single integrations confirmed by Southern blot analysis.

Complementation of each RasGEF deletion was achieved by targeted, ectopic integration of each gene under the control of the endogenous promoter using a previously described CRISPR/Cas9 technique (Al Abdallah, Ge, & Fortwendel, 2017). Briefly, to generate the repair templates for complementation, the coding region plus 1 kb of native promoter for each RasGEF was PCR amplified from genomic DNA and fused to a phleomycin resistance cassette, previously amplified from the plasmid pAGRP (Fortwendel et al., 2012), using an overlap‐extension PCR technique (Szewczyk et al., 2006). Repair templates were amplified with primers designed to incorporate microhomology regions with 35 bp of homology to the target locus. Transformation was carried out as previously described (Al Abdallah et al., 2017). Briefly, a crRNA was designed to target a selected protospacer sequence inside the endogenous, non‐functional A. fumigatus *pyrG* locus of each RasGEF mutant. The guide RNA (gRNA) was generated by mixing equal molar amounts of crRNA and tracrRNA in nuclease‐free duplex buffer and boiling at 95°C for 5 min. This mixture was allowed to cool at room temperature for 10 min before 1.5 μl of gRNA were combined with 1.5 μl of commercially available Cas9 (1 μg/μl) and 22.5 μl of Cas9 working buffer (20 mM HEPES, 150 mM KCl, pH 7.5). This was followed by incubation at room temperature for 5 min to allow formation of the ribonucleoprotein complex and then used for protoplast transformation. Correct integration of the repair templates at the endogenous, non‐functional *pyrG* locus was confirmed by PCR and Southern blot.

To localise RasA in the *ΔgefA* and *ΔgefB* strains*,* the GFP‐RasA expression vector (Fortwendel et al., 2012) was first employed to amplify a repair template for transformation into each deletion background. Two protospacer adjacent motifs were chosen, one upstream and one downstream of the RasA coding sequence, two gRNAs were designed to guide Cas9 mediated double‐strand DNA breaks, and transformation was carried out as previously described (Al Abdallah et al., 2017). GFP fluorescence of resulting transformants was analysed using a Nikon Ni‐U fluorescence microscope, equipped with a GFP filter.

A mutant strain with regulatable SH3‐class RasGEF activity was generated by using the tetracycline inducible promoter system (TetOn) to control *gefB* expression in the background of the *ΔgefA* strain, using previously described CRISPR‐Cas9 techniques (Al Abdallah et al., 2017). Briefly, the TetOn cassette and adjacent pyrithiamine resistance gene was PCR‐amplified from plasmid pCH008 (a generous gift from Sven Krappmann; Erlangen, Germany) using primers containing 35 bp microhomology region (+2 bp relative to the start codon). Transformation was carried out as previously described (Al Abdallah et al., 2017) and protoplasts were plated onto sorbitol minimal medium containing pyrithiamine (500 ng/ml) and doxycycline (30 μg/ml). Pyrithiamine‐resistant colonies were screened by PCR and by Southern blot to ensure single‐copy, homologous integration.

In a similar way, we generated a regulatable Ras activity mutant strain. In this case, the pyrithiamine resistance cassette from pCH008 plasmid was replaced by a phleomycin resistance cassette to generate the plasmid pCH008‐phleo. This plasmid was used to amplify the TetOn promoter adjacent to the phleomycin cassette and replace the RasA promoter in the background of the Δ*rasB* with the same CRISPR/Cas9 technique described above.

### 4.4 β‐glucan immunostaining and content analysis

To determine possible cell wall changes in the Δ*gefA* strain, 2 × 10^7^ conidia from both control and Δ*gefA* strains were incubated in GMM with agitation for 9 and 10.5 hr, respectively, at 37°C. The germlings were washed twice with dH_2_O and once with PBS and incubated for 1 hr at 4°C with 10 μg/ml of Fc‐hDectin‐1a (Invivogen) in PBS. After this time, the fungal material was washed three times with PBS and then incubated at 4°C for one additional hour with a FITC‐conjugated anti‐human IgG secondary antibody (ThermoFisher, Waltham, Massachusetts; diluted 1:200 in PBS). Next, the samples were washed in order to remove unbound antibodies and observed (Nikon Ni‐U) using GFP filter.

1,3‐β‐D‐glucan quantification was performed as previously described (Fortwendel et al., 2009). Briefly, 10^7^ conidia from both control and Δ*gefA* strains were inoculated in yeast glucose medium and incubated at 37°C for 18 hr with agitation. The hyphae were then washed three times with 0.1 M NaOH and lyophilized. Next, 5 mg of hyphal tissue were homogenised and incubated with 250 μl of 1 M NaOH at 52°C for 30 min. After incubation, 50 μl of each sample were mixed with 185 μl of aniline blue mix (183 mM glycine, 229 mM NaOH, 130 mM HCl, 618 mg/l aniline blue, pH 9.9) in a masked 96‐well plate and incubated at 52°C for additional 30 minutes. After the plate was allowed to reach room temperature, fluorescence was read at 405 nm excitation and 460 nm emission and the values were normalised to a Curdlan (Sigma) standard curve. Three biological replicates were performed containing each one three technical replicates. The amount of 1,3‐β‐D‐glucan was compared between strains using Student's t‐test (GraphPad v7).

### 4.5 Western blot analysis

All strains were grown in GMM + YE overnight at 37°C with agitation at 250 rpm. The resulting fungal mass was filtered and rinsed with sterile, deionised water and crushed with liquid nitrogen. The macerated hyphal material was resuspended in 1:1 volumes of extraction buffer (25 mM Tris–HCl [pH 7.5], 10 mM MgCl_2_,150 mM NaCl, 1 mM EDTA, 0.01% NP‐40, 2% glycerol, 1 mM Pefabloc [Sigma], 1 mM protein inhibitor cocktail [Sigma]), and the resulting crude lysates were centrifuged at 3,500 rpm for 9 min at 4°C. Cleared lysates were then transferred to new tubes and the total protein concentration was quantified using the Bradford assay. Fifty micrograms of cleared lysate were boiled before SDS‐PAGE separation. Membranes were probed with the anti‐Ras, clone RAS10 mouse monoclonal primary antibody (1:2,000 dilution, EMD Millipore) and followed by the secondary antibody, a horseradish peroxidase‐conjugated goat antimouse IgG2a (1:2,000 dilution, Abcam). Blots were imaged using a Bio‐Rad ChemiDoc XRS HQ System and QuantityOne software (v4.6.5, Bio‐Rad). The assay was performed in biologic triplicate for each strain.

### 4.6 Analysis of *rasA* expression levels

All strains were grown in GMM + YE at 37°C/250 rpm for 18 hr. After incubation, extraction of total RNA was performed using the Qiagen RNEasy Mini Kit, following manufacturer's recommendations. Six micrograms of total RNA from each sample were digested with RNase‐free Turbo DNase (Invitrogen, Carlsbad, California) following the manufacturer's protocol. Next, cDNA was synthesised using the SuperScript II system (Invitrogen), according to manufacturer's instructions. Quantitative reverse transcription PCR was performed using SYBR® Green Master Mix (Bio‐Rad, Hercules, California) in a CFX Connect Real‐Time System (Bio‐Rad). RasA and β‐tubulin (TubA) specific qPCR primers were designed to flank introns, where possible. Transcript levels were calculated by comparative ΔCt and normalised to β‐tubulin. Each experiment was performed in biological triplicate.

### 4.7 Murine models of invasive pulmonary aspergillosis

All the studies were performed in accordance to approved protocols by the IACU committee of the University of Tennessee Health Science Center. Groups of 10 female CF‐1 mice (Charles River or Envigo), weighing approximately 24 g, were used for each experimental arm in this study. In order to observe possible differences in pathogenicity, we performed two models of IA with different immunosuppressive regimens. For the non‐neutropenic model, animals were immunosuppressed with 40 mg/kg of triamcinolone acetonide (Kenalog, Bristol‐Myers Squibb, New York City, New York), given subcutaneously 1 day before the infection. To induce neutropenia as a second model, in addition to the single triamcinolone injection, mice received intraperitoneal injections of cyclophosphamide (Sigma‐Aldrich; 150 mg/kg), beginning on day 3 before infection and on every third day thereafter for a total of 3 to 5 injections, depending on the health of the animal.

On the day of the infection, mice were anaesthetised with 5% isoflurane and challenged by nasal instillation with 1 × 10^5^ conidia of each strain. Mice were monitored twice daily for 15 days and were euthanized by anoxia with CO_2_ at the end of the experiment or when moribund. Pairwise survival comparisons of all treatment groups versus the control strain were performed by Kruskal‐Wallis (nonparametric one‐way analysis of variance) analysis with Dunn's posthoc testing (GraphPad Prism v7).

In addition to the survival studies, groups of two mice per strain were immunosuppressed and infected as described above and euthanized 3 days postinfection for histopathology analyses. Whole lungs were fully inflated by intratracheal perfusion with 10% buffered formalin neutral solution. Routine histological techniques were used to prepare paraffin‐embedded tissue, and 5 μm sections of the superior, middle, and inferior lobes of the right lung were stained with haematoxylin and eosin, and Grocott's methenamine silver.

## CONFLICTS OF INTEREST

The authors declare that they have no competing interests.

## FUNDING INFORMATION

This work was supported by NIH grants R01 AI106925 and R21 AI139388.
